# Enhancing active aging through exercise: a comparative study of high-intensity interval training and continuous aerobic training benefits

**DOI:** 10.3389/fragi.2025.1493827

**Published:** 2025-06-02

**Authors:** Federico Zoila, Francesca Martina Filannino, Maria Antonietta Panaro, Italo Sannicandro, Antonia Cianciulli, Chiara Porro

**Affiliations:** ^1^ Department of Clinical and Experimental Medicine, University of Foggia, Foggia, Italy; ^2^ Department of Neurosciences, Biomedicine and Movement Sciences, University of Verona, Verona, Italy; ^3^ Department of Biosciences, Biotechnologies and Environment, University of Bari, Bari, Italy

**Keywords:** active aging, high-intensity interval training, continuous aerobic training, older adults, physical fitness

## Abstract

**Introduction:**

As global life expectancy increases, the importance of maintaining health and functional independence in older adults becomes paramount. This study investigates the comparative effects of High-Intensity Interval Training (HIIT) and Continuous Aerobic Training (CAT) on physical fitness, cognitive function, and overall wellbeing in older populations.

**Methods:**

This review analyzed randomized controlled trials (RCTs) focusing on older adults (aged 60–85) engaged in High-Intensity Interval Training (HIIT) and Continuous Aerobic Training (CAT). Key metrics included cardiovascular fitness, measured via VO2 max tests and 6-minute walk tests; muscle strength, assessed using handgrip dynamometry and sit-to-stand tests; and cognitive performance, evaluated with Montreal Cognitive Assessment (MoCA) and Trail Making Tests (TMT). Mental health was assessed using the Beck Depression Inventory (BDI), and quality of life was measured with the SF-36 Health Survey. A total of 18 RCTs were included.

**Results:**

Across the reviewed studies, both HIIT and CAT produced significant health benefits in older adults. HIIT led to a 15%–20% increase in VO2 max, a 12% improvement in muscle strength, and a 10%–15% enhancement in cognitive function, particularly in memory and executive tasks (MoCA scores). HIIT also reduced fall risk by 23%, likely due to its impact on dynamic balance and coordination. CAT, meanwhile, improved aerobic capacity by 10%–15% and was particularly effective in reducing depressive symptoms by 5%–10%, as measured by the Beck Depression Inventory (BDI). CAT also showed moderate benefits for mental wellbeing and mood regulation.

**Discussion:**

While both HIIT and CAT show significant short-term benefits, their long-term effects need more exploration. HIIT has demonstrated sustained improvements in VO2 max and cognitive function for up to 6 months, but its long-term impact on age-related decline is unclear. CAT offers lasting benefits for aerobic capacity and mental health, though more data are needed on its effect on long-term functional independence. Future research should focus on longitudinal studies to assess the durability of these benefits and explore combining HIIT and CAT for optimal outcomes. Additionally, using wearable technology to track adherence and progress could provide valuable insights.

## 1 Introduction

The demographic transition is one of the most challenging developments in our modern world since human life expectancy has substantially grown worldwide (Flatt and Partridge, 2018). Indeed, over the past decades, medical progress has significantly increased life expectancy. Thus, more than 2 billion individuals are expected to be older than the age of 60 by 2050 (Mahindru et al., 2023). In Western countries, life expectancy has increased by about 20 years since 1950 (Dattani et al., 2023), and almost 10% of people living in developed countries will be 80 years old or older by 2050 (Harper, 2014). Nevertheless, a substantial gap exists between total and healthy life expectancy (Seals et al., 2016), meaning people live several years with functional limitations. This gap, which burdens individuals, their families, and society due to increased health costs (Harper, 2014), has multidimensional causes and may partly depend on biological aging mechanisms and lifestyle behavior, such as low physical activity and high sedentary time (Raffin et al., 2023).

Most researchers concur that aging is a natural, physiological process of growing older experienced by every individual at varying rates (Grayston, 2018). Due to the diversity in aging patterns, it is crucial to differentiate between chronological and biological age. Chronological age denotes only the time since birth, while biological age encompasses a wide range of physical, physiological, and cognitive functions influenced by molecular and cellular processes (Fuellen et al., 2019). Biological age can provide a more accurate prediction of health outcomes, such as hospital mortality, compared to chronological age. For instance, a study found that patients with a biological age significantly older than their chronological age had a higher risk of mortality (Ho et al., 2023). The “age gap,” the difference between biological and chronological age, is a complementary indicator of aging, revealing insights into individual health risks (Salih et al., 2023). While biological age offers a nuanced understanding of aging, it is essential to recognize that both measures can be influenced by lifestyle and health interventions, complicating the aging narrative. The exact biology of aging remains a topic of debate, making it difficult to establish a universally accepted definition of normal aging (Cohen et al., 2020). Aging causes tissues and organs to operate less well and lose physiological integrity with time. Human lifespans are getting longer, and aging also gets older. Three stages of aging have recently been identified: early or elderly old age, senile or middle-aged old age, and late old age, also known as long-liver (Dodig et al., 2019). One could say that the age/aging phases are easy to recognize, but the mechanisms responsible for the aging process are complex to define and harder to prove. The natural aging process affects all organisms. Time, hereditary, and, to a greater extent, environmental variables contribute to the complicated biological aging process. It happens in different ways in different cells and tissues. The biological age does not always correspond to the chronological age because people age at different rates. The human body exhibits numerous markers and aging-related alterations. A few categories can be used to categorize the changes that come with aging: normal aging, physical illnesses and some chronic issues, and psychological, cognitive, and social changes. (Jaul and Barron, 2017).

The changes could be physiological, such as the decline in muscle mass and strength, a condition known as sarcopenia that can lead to decreased mobility and increased frailty in older adults. Additionally, there is a reduction in bone density, which can result in an increased risk of fractures and osteoporosis (Larsson et al., 2019). Aging also brings about changes in cognitive function. Many individuals experience a decline in memory, processing speed, and executive function as they age. These changes can impact daily activities and independence, making it essential to develop strategies for maintaining cognitive health (Erickson et al., 2019; Feng et al., 2022). Another significant effect of ageing is the heightened susceptibility to chronic diseases. Conditions such as heart disease, diabetes, and cancer become more prevalent as individuals grow older (Maresova et al., 2019).

Aging is the primary risk factor for the majority of prevalent chronic diseases, such as dementia, cardiovascular disease, and cancer (Niccoli and Partridge, 2012; Zhang et al., 2020). As a result, the proportion of persons with one or more chronic diseases rises (Fiacco et al., 2019). Aging and disease are not the same things, even though it is a significant risk factor for many chronic illnesses. If aging is a natural and inevitable process that occurs in all living organisms, including humans and refers to the gradual loss of physiological function over time, resulting in physical, cognitive, and emotional changes, healthy aging, on the other hand, refers to maintaining a high level of functioning and wellbeing as one age. This includes maintaining physical fitness, cognitive abilities, and emotional health. Healthy aging looks back on a long historical development with concepts like successful, healthy, productive, or active aging (Behr et al., 2023). Successful aging, also known as optimal aging, takes the concept of healthy aging a step further. It involves maintaining a high level of functioning and finding satisfaction, purpose, and fulfillment in later years (Mackenzie, 2012). Understanding the factors contributing to these conditions and implementing preventive measures is crucial for promoting healthy aging.

Physical activity and exercise are crucial in promoting healthy and successful aging. Regular physical activity can help mitigate the decline in muscle mass and strength, combatting the effects of sarcopenia and promoting better mobility and independence in older adults. Additionally, exercise, particularly weight-bearing and resistance training, can contribute to maintaining bone density and reducing the risk of fractures and osteoporosis. Furthermore, physical activity has positively impacted cognitive function, including improved memory, attention, and executive function. This can significantly contribute to maintaining independence and quality of life as individuals age (Erickson et al., 2019; Faienza et al., 2020).

In terms of chronic disease prevention, participating in regular exercise has been associated with a reduced risk of conditions such as heart disease, diabetes, and certain types of cancer. Through its influence on weight management, blood pressure regulation, and insulin sensitivity, physical activity can help mitigate the risk factors for these diseases, thereby promoting overall health and wellbeing as individuals age (Ruegsegger and Booth, 2018).

Moreover, staying physically active also contributes to emotional and psychological wellbeing, promoting a sense of purpose, satisfaction, and fulfillment in later years. Integrating physical activity and exercise into daily routines supports active and healthy aging (Rebelo-Marques et al., 2018; Szychowska and Drygas, 2022).

## 2 Dimensions of aging: active, healthy and successful

Active, healthy, and successful aging are interrelated concepts that have gained significant attention in gerontology and public health research. While these terms are often used interchangeably, they encompass distinct dimensions of the aging process. According to Rowe and Kahn (2015), active aging refers to the process of optimizing opportunities for health, participation, and security to enhance the quality of life as people age. This perspective emphasizes the importance of maintaining engagement in various domains of life, including physical, social, and cognitive activities, to promote wellbeing in later years (Delle Fave et al., 2018). Pivotal studies have identified several specific metrics that can effectively measure active aging, focusing on health, social participation, and environmental factors. Many tools emphasize the importance of physical and mental health as foundational elements of active aging. The Active Aging Index, for instance, incorporates health status as a primary metric (Punyakaew et al., 2023). Engagement in social activities is crucial. Marsillas Rascado et al. developed a measurement tool that includes social participation as a core component, highlighting its role in individual wellbeing (Marsillas Rascado et al., 2024). The active aging framework also stresses the need for a secure and enabling environment. Punyakaew et al. identified these factors essential for fostering active aging among older adults (Punyakaew et al., 2023). Moreover, The Active Aging Scale and the Active Aging Index have been validated for their psychometric properties, ensuring their reliability in various contexts (Han et al., 2023). While these metrics provide a robust framework for assessing active aging, there is ongoing debate about the need for tools tailored to specific subgroups of older adults, suggesting that more than a one-size-fits-all approach is needed (Xiao et al., 2024). Healthy aging, on the other hand, focuses more specifically on the preservation of physical and mental health throughout the aging process. As defined by the World Health Organization, healthy aging involves “developing and maintaining the functional ability that enables wellbeing in older age.” This encompasses not only the absence of disease or disability but also the ability to adapt and cope with the changes and challenges that come with aging (Briggs et al., 2016). Fundamental studies highlight several specific metrics that can effectively measure healthy aging, emphasizing the need for comprehensive approaches. Healthy Aging Index integrates multiple dimensions of health, including physical, psychological, and social factors, to provide a holistic view of healthy aging (Behr et al., 2023). Research has shown that analyzing community-level data can reveal disparities in healthy aging, with factors such as chronic diseases and disabilities being critical indicators (Dugan et al., 2022). Furthermore, the World Health Organization emphasizes the importance of maintaining functional ability as a core aspect of healthy aging, which includes mobility, social engagement, and the ability to meet basic needs (Sadana and Banerjee, 2019). While these metrics provide valuable insights, the complexity of healthy aging necessitates ongoing research to refine definitions and measurement tools, ensuring they are applicable across diverse populations and contexts. Successful aging represents another perspective on aging, emphasizing subjective wellbeing and life satisfaction in older adults. Baltes and Baltes (2018) proposed that successful aging involves “optimizing gains and minimizing losses in the physical, cognitive, and socioemotional domains of functioning.” This framework recognizes that successful aging is not solely determined by objective health indicators but also by individual perceptions and experiences of aging (Baltes and Smith, 2003). Key studies have identified several dimensions contributing to understanding and measuring successful aging. Multidimensional Successful Aging Scale (MSAS) includes nine factors such as adaptive coping, social contribution, and positive attitudes, demonstrating the complexity of successful aging beyond traditional models (Chung and Yeung, 2022). Also, factors like metabolic health, adherence to healthy diets, and physical activity are crucial for successful aging (Rodrigues et al., 2023). Measurement Approaches like the Multi-Domain Responder Index (MDRI) integrate various functional, social, and cognitive outcomes to create a comprehensive measure of successful aging, addressing the heterogeneity in older adult populations (Schönstein, 2024). Moreover, research indicates that maintaining chronic disease management, employment, and social engagement are vital for sustaining successful aging over time (Seong et al., 2024). While these metrics provide a robust framework for understanding successful aging, it is essential to consider the individual variability in aging experiences, which may challenge the universality of these indicators. In essence, while active aging emphasizes participation, healthy aging focuses on health preservation, and successful aging encompasses subjective wellbeing; together, these concepts provide a comprehensive understanding of aging that extends beyond mere longevity to encompass various dimensions of wellbeing and fulfillment in later life. Table 1 summarizes the different types of aging.

**TABLE 1:** Dimensions of aging.

Type of ageing	Definition	Key characteristics	Key references
Active Ageing	Optimising opportunities for health, participation, and security to enhance quality of life as people age	-Emphasis on engagement in physical, social, and cognitive activities - Promotes wellbeing in later years	Rowe and Kahn (2015), Delle Fave et al. (2018)
Healthy Ageing	Developing and maintaining the functional ability that enables wellbeing in older age	- Focus on the preservation of physical and mental health - Includes absence of disease or disability - Ability to adapt and cope with ageing challenges	Briggs et al. (2016)
Successful Ageing	Optimising gains and minimising losses in physical, cognitive, and socioemotional domains of functioning	-Emphasises subjective wellbeing and life satisfaction -Not solely determined by objective health indicators -Includes individual perceptions and experiences of ageing	Baltes and Baltes (2018), Baltes and Smith (2003)

Recent research has further elucidated the complex interplay between these dimensions of aging and their implications for older adults’ overall wellbeing. For instance, a study by Steptoe and colleagues (Steptoe et al., 2015) examined the association between different aspects of active aging, such as physical activity, social engagement, cognitive stimulation, and subjective wellbeing in a large sample of older adults (Steptoe and Fancourt, 2020). They found that individuals engaged in various activities across these domains reported higher life satisfaction and positive affect levels. Similarly, a longitudinal study by Rowe and Kahn (2015) explored healthy aging trajectories among older adults over a 10-year period, identifying factors such as physical fitness, cognitive function, and social support as predictors of successful aging outcomes, including longevity and quality of life. These findings underscore the importance of adopting a multidimensional approach to aging that addresses physical health, social connectedness, cognitive vitality, and emotional wellbeing.

Moreover, recent advancements in technology and healthcare have presented new opportunities for promoting active, healthy, and successful aging. Interventions leveraging digital health tools, such as wearable activity trackers, mobile health apps, and telemedicine platforms, have shown promise in supporting older adults’ efforts to maintain physical activity levels, manage chronic conditions, and access healthcare services remotely (Abadir et al., 2023; Li et al., 2024). Additionally, community-based programs promoting social inclusion and lifelong learning have effectively fostered social engagement and cognitive stimulation among older adults (Chang et al., 2020). By harnessing these innovative approaches, policymakers, healthcare providers, and community organizations can facilitate aging in place and empower older adults to age with dignity and independence.

Physical exercise emerges as a cornerstone in the pursuit of optimal aging. Research consistently highlights the profound impact of regular physical activity on various dimensions of wellbeing in later life. By engaging in regular exercise, older adults can maintain physical fitness and prevent age-related decline, bolster cognitive function, promote social interaction, and enhance emotional resilience. As such, prioritizing physical activity promotion initiatives becomes imperative for fostering active aging, promoting healthy aging, and facilitating successful aging outcomes.

In this review, we will analyze the impact of High-Intensity Interval Training (HIIT) and Continuous Aerobic Training (CAT) on aging, trying to understand, through the common points and differences, which is the right methodology to practice for an elderly person.

## 3 High-intensity interval training: a promising exercise regimen for enhancing health and cognitive function in older adults

High-Intensity Interval Training (HIIT) is a form of cardiovascular exercise characterized by alternating short periods of intense anaerobic exercise with less intense recovery periods. This type of training can include various activities such as sprinting, cycling, or bodyweight exercises. The structure of a typical HIIT workout involves brief, high-intensity efforts lasting from 20 s to a few minutes, followed by equally short or slightly longer recovery phases. The total duration of a HIIT session can range from 10 to 30 min, making it an efficient workout choice for those with limited time.

The popularity of HIIT has surged due to its efficiency and effectiveness in improving overall health and fitness. Research has shown that HIIT can significantly enhance cardiovascular and metabolic health, often in a fraction of the time required for traditional steady-state exercise. For example, a study by Gillen and Gibala (2014) demonstrated that a 10-min HIIT session, which included only 1 minute of intense exercise, produced similar benefits to a 50-min session of moderate-intensity continuous training. This efficiency is particularly appealing to individuals seeking to maximize health benefits without investing large amounts of time (Gillen and Gibala, 2014).

One of the primary mechanisms by which HIIT improves fitness is its impact on mitochondrial function and oxidative capacity. Bartlett et al. (2011) found that HIIT promotes mitochondrial biogenesis, enhancing the muscle cells’ ability to produce energy. This adaptation improves aerobic capacity and increases energy usage efficiency during exercise, aiding in weight management and overall physical performance (Bartlett et al., 2011).

In addition to its physiological benefits, HIIT is also known for its psychological advantages. The varied nature of HIIT workouts can make them more engaging and enjoyable compared to monotonous steady-state cardio exercises. Thum et al. (2017) reported that participants found HIIT to be more enjoyable and reported higher exercise adherence levels than traditional moderate-intensity continuous training. This enjoyment factor can play a crucial role in maintaining long-term exercise habits, which are essential for sustained health benefits (Thum et al., 2017).

HIIT is highly adaptable and can be customized to suit different fitness levels and preferences. It can be performed using minimal or no equipment, making it accessible to a wide range of people. Beginners can start with lower intensity and gradually increase the difficulty as their fitness improves. This scalability makes HIIT an inclusive and versatile training method. Recent research supports its advantages, solidifying HIIT’s role as a cornerstone in contemporary fitness programs.

HIIT has gained attention in recent years due to its effectiveness in improving health and exercise performance across various populations, including older adults. Recent studies highlight that HIIT can significantly enhance cardiorespiratory fitness and overall health and reduce body fat levels in older individuals (Atakan et al., 2021; Engel et al., 2018). This training model, characterized by short bursts of intense physical activity followed by brief rest periods or lower-intensity exercise, has been extensively studied, revealing significant positive effects on various aspects of aging, including cardiovascular health (Weston et al., 2014). HIIT has been shown to improve cardiac output, vascular endothelial function, and aerobic and anaerobic capacity, making it a highly effective exercise modality for enhancing overall cardiovascular health in older adults (Milanović et al., 2015). Studies have demonstrated that HIIT leads to improvements in cardiorespiratory fitness, exercise tolerance, blood pressure, lipid profiles, and vascular reactivity in older adults, ultimately reducing the risk of dependency, cognitive impairment, and premature mortality (Sian et al., 2022). Additionally, HIIT has been found to increase muscle microvascular blood flow, improve endothelial function, and enhance muscle capillarization, all of which are crucial for mitigating age-related declines in cardiovascular function and combating conditions like sarcopenia and cardiovascular disease (Herrod et al., 2021). Overall, HIIT emerges as a time-efficient and effective intervention for promoting cardiovascular health in seniors, offering a promising approach to enhancing their physiological resilience and overall wellbeing.

In addition to cardiovascular benefits, HIIT significantly increases muscle mass and strength, counteracting the age-related decline in muscle mass known as sarcopenia (Borde et al., 2015). The intermittent high-intensity activity promotes anabolic responses in muscle tissue, leading to more significant muscle hypertrophy and strength gains than traditional endurance training (Fyfe et al., 2014). Cognitive function is another area where HIIT demonstrates remarkable benefits. Emerging evidence suggests that HIIT can enhance cognitive abilities such as memory, attention, and executive functions in older adults, mediated by increased Brain-Derived Neurotrophic Factor (BDNF) levels, improved cerebral blood flow, and reduced inflammation (Heisz et al., 2016).

HIIT plays a crucial role in enhancing cognitive function in seniors by promoting neuroplasticity and neurogenesis. Research indicates that HIIT can increase BDNF levels in various brain areas, including the hippocampus, spinal cord, cerebellum, and cortical regions, leading to improved cognition (Sabita et al., 2023). HIIT has been shown to improve executive function in older adults, with results comparable to those of young, healthy adults, highlighting its potential to combat cognitive decline associated with aging (Mekary et al., 2022). Additionally, a study on elderly individuals with mild cognitive impairment demonstrated that a HIIT program significantly improved cognitive functions such as attention, verbal fluency, and concentration, emphasizing the positive impact of HIIT on cognitive health in the geriatric population (Rivas-Campo et al., 2023). Furthermore, HIIT may help synchronize disrupted sleep-wake cycles, improve sleep quality, and potentially delay cognitive decline associated with aging, further emphasizing its role in enhancing cognitive function in seniors (Garo-Pascual et al., 2023). These findings underscore the importance of HIIT as a promising non-pharmacological approach to enhance cognitive function in seniors and mitigate age-related cognitive decline.

HIIT shows promise in improving balance and coordination in seniors, although the evidence is mixed. Studies suggest that HIIT can enhance lower limb strength, dynamic balance, and subjective balance perception in older adults, making it a valuable supplement to existing fall prevention programs (Elboim-Gabyzon et al., 2021). Additionally, HIIT has been found to be a safe and effective training method for seniors, leading to improvements in cardiovascular, pulmonary, hemodynamic, lipid, muscle, and cognitive functions (Gomez Piqueras and Sanchez Gonzalez, 2019). However, research also indicates that aging may lead to changes in neuromuscular coordination patterns during quiet standing, with seniors exhibiting altered ankle muscle coordination and increased co-activation compared to young adults, although these patterns were not significantly affected by acute HIIT sessions (Donath et al., 2015). Therefore, while HIIT can benefit seniors’ overall health and physical performance, its direct impact on balance and coordination may vary and require further investigation.

HIIT has been shown to impact metabolic health across various populations significantly. Studies have demonstrated that HIIT can improve glycolipid metabolism in children with metabolic disorders, leading to beneficial changes in triglyceride, cholesterol, glucose, and insulin levels (Mekari et al., 2020; Men et al., 2023). Additionally, HIIT has been found to enhance liver metabolism, reduce inflammation, and improve insulin signaling pathways in type 2 diabetes mellitus mice, indicating its effectiveness in addressing lipid metabolism disorders and inflammation in the liver (Hu et al., 2023; Zheng et al., 2020). Furthermore, HIIT has been shown to improve cardiometabolic health in overweight/obese individuals, promoting positive adaptations in cardiorespiratory fitness, body composition, blood pressure, glucose metabolism, and lipid profiles, with longer-lasting effects compared to moderate-intensity continuous training (Bo et al., 2023). HIIT has been shown to enhance glycolipid metabolism, contributing to reductions in critical markers such as triglycerides, cholesterol, glucose, and insulin levels. For elderly individuals who are at a higher risk for metabolic disorders such as type 2 diabetes, these improvements are particularly crucial. Recent studies have demonstrated that HIIT significantly improves insulin sensitivity and reduces fasting glucose levels in elderly participants. For instance, research by Wewege et al. (2018) found that older adults participating in a 12-week HIIT program experienced a 10%–15% reduction in Homeostatic Model Assessment of Insulin Resistance (HOMA-IR), a widely used marker of insulin resistance, compared to those performing moderate-intensity continuous training (MICT) (M. A. Wewege et al., 2018). Additionally, studies have shown a 5%–10% reduction in fasting blood glucose levels among elderly participants, underscoring HIIT’s potential to improve long-term glycemic control (Cassidy et al., 2017). Furthermore, HIIT has been linked to favorable changes in lipid profiles in elderly populations, including a 5%–20% reduction in triglycerides and LDL cholesterol, critical markers for cardiovascular health and metabolic syndrome. These changes enhance metabolic health and reduce the risk of cardiovascular events and mortality in older adults (Bo et al., 2023; Hu et al., 2023). Given the time-efficient nature of HIIT, these metabolic improvements can be achieved with minimal weekly exercise duration, making it a feasible and effective intervention for elderly individuals who may face barriers to longer exercise sessions. HIIT emerges as a time-efficient and effective intervention for enhancing metabolic health by targeting various aspects of glycolipid metabolism, inflammation, and insulin sensitivity. These findings suggest that incorporating HIIT into regular physical activity routines for older adults can play a critical role in improving glucose metabolism and overall cardiometabolic health, reducing the risk of age-related metabolic diseases.

HIIT offers several key benefits for seniors. Research indicates that HIIT can improve sleep quality, reduce sleep onset latency, and lower wake after sleep onset, potentially delaying cognitive and physical decline in older adults (Coswig et al., 2020). In elderly women, HIIT has been shown to significantly enhance body composition, insulin resistance, blood lipids, functional capacity, cardiorespiratory fitness, and quality of life compared to moderate-intensity continuous training (MICT) (Rohmansyah et al., 2023). Studies on healthy older adults have demonstrated that HIIT significantly improves sleep quality, fatigue, body composition, strength, anabolic hormones, blood lipids, VO2 max, exercise tolerance, and systolic blood pressure, especially when combined with nutritional support (Labrin et al., 2023). Additionally, HIIT can potentially improve muscle mass/strength, maximal oxygen consumption (VO2 max), and cognitive abilities in older adults, making it a valuable exercise regimen for this demographic (Ito, 2022a). Moreover, HIIT has been found to modulate immunological parameters without increasing the incidence of upper respiratory tract infections, suggesting its safety and potential benefits for older adults’ immune function (Scartoni et al., 2023).

HIIT has shown promise in managing chronic conditions in seniors by improving functional movement, cardiorespiratory fitness, and overall health awareness (Juan and Xianyi, 2023; Keating et al., 2020; Stern et al., 2023). Research indicates that HIIT interventions can effectively enhance functional movement and cardiorespiratory fitness in older adults, potentially reducing the risk of chronic diseases such as hypertension, diabetes, and chronic respiratory conditions (Juan and Xianyi, 2023; Keating et al., 2020; Stern et al., 2023). HIIT has been found to be well-tolerated and beneficial for older adults, with studies highlighting its positive impact on various health indicators, including blood glucose, blood pressure, and body mass index, ultimately leading to improved quality of life and self-management efficacy (Juan and Xianyi, 2023; Marriott et al., 2021). However, further research is needed to explore the specific effects of HIIT on seniors with multiple complex chronic conditions, emphasizing the importance of individualized functional programs and patient safety monitoring (Brockway et al., 2023).

HIIT can be safe for seniors with chronic conditions, but safety considerations must be prioritized (Brockway et al., 2023; Ito, 2022b). Research indicates that HIIT interventions in older adults may effectively improve functional movement, though the superiority of HIIT over moderate-intensity continuous training (MICT) remains inconclusive (Stern et al., 2023). While HIIT is a popular and time-efficient exercise method that can enhance aerobic capacity and muscle strength in various populations, including older adults (Ito, 2022a), the tolerability and effects in seniors are less well-known. Studies have shown that HIIT protocols are diverse, generally well-tolerated, and may offer numerous health benefits to older adults, but further research, especially in clinical populations representative of seniors with chronic conditions, is needed to evaluate the full extent of its safety and efficacy in this specific demographic (Marriott et al., 2021).

The effects of HIIT on balance and coordination in older adults are critical. Research shows HIIT significantly enhances dynamic balance and muscle strength, both crucial for reducing fall risk. For instance, Elboim-Gabyzon et al. (2021) reported that older adults engaging in HIIT improved balance scores by 10% on the Berg Balance Scale and TUG performance by 12% compared to controls after 12 weeks (Elboim-Gabyzon et al., 2021). Similarly, Herrod et al. (2021) found that HIIT led to an 11% gain in lower limb strength and a 10% increase in gait speed, contributing to enhanced mobility and stability (Herrod et al., 2021). These results suggest that incorporating HIIT into fall prevention programs for older adults can lead to better balance and strength outcomes, ultimately reducing the risk of falls and enhancing overall mobility and independence.

HIIT has shown promising results in improving various aspects of health in older adults, including muscle strength, cognitive abilities, and fall risk factors. While there is a lack of consensus on specific HIIT routines recommended for seniors due to the diversity in protocols and outcomes across studies (Marriott et al., 2021), a combination of aerobic and strength training has been suggested as an effective exercise program for older adults to enhance both muscle mass/strength and maximal oxygen consumption (VO2max) (Falck et al., 2017). HIIT has been increasingly recognized as a time-efficient way to improve VO2 max and muscle strength/power in older adults. However, the adaptation and safety considerations for this population are still being explored (Blackwell et al., 2021). Further research is needed to establish more tailored and standardized HIIT protocols for seniors, considering factors like age, physical function, safety, and adherence to participation rates (Pires Peixoto et al., 2020). Incorporating HIIT into physical activity promotion initiatives for older adults can yield comprehensive benefits, encompassing improved cardiovascular health, increased muscle strength, enhanced cognitive function, and overall wellbeing. The growing body of research underscores HIIT’s promise to empower older adults to maintain their physical and cognitive vitality, ultimately enhancing their quality of life and independence. These findings highlight HIIT as a valuable tool for promoting healthy aging and overall wellbeing in the aging population (Keating et al., 2022). Table 2 summarizes the benefits of HIIT on aging.

**TABLE 2:** HIIT benefits on aging.

Benefit	Description	References
Cardiovascular and Metabolic Health	Improves cardiovascular health, enhances metabolic function, reduces body fat levels, improves cardiac output, vascular endothelial function, aerobic and anaerobic capacity	Gillen and Gibala (2014), Weston et al. (2014), Milanović et al. (2015), Sian et al. (2022), Herrod et al. (2021), Atakan et al. (2021), Engel et al. (2018)
Mitochondrial Function	Promotes mitochondrial biogenesis, enhancing muscle cells’ ability to produce energy, improving aerobic capacity and energy usage efficiency	Bartlett et al. (2011)
Exercise Enjoyment and Adherence	More engaging and enjoyable than steady-state cardio, leading to higher exercise adherence levels	Thum et al. (2017)
Muscle Mass and Strength	Increases muscle mass and strength, counteracting age-related decline in muscle mass (sarcopenia), promotes anabolic responses in muscle tissue, leads to significant muscle hypertrophy and strength gains	Borde et al. (2015), Fyfe et al. (2014)
Cognitive Function and Brain Health	Enhances cognitive abilities such as memory, attention, and executive functions, increases Brain-Derived Neurotrophic Factor (BDNF) levels, improves cerebral blood flow, reduces inflammation, promotes neuroplasticity and neurogenesis, improves sleep quality	Heisz et al. (2016), Sabita et al. (2023), Mekary et al. (2022), Rivas-Campo et al. (2023), Garo-Pascual et al. (2023), Coswig et al. (2020), Labrin et al. (2023)
Metabolic Health	Improves glycolipid metabolism, reduces triglyceride, cholesterol, glucose, and insulin levels, enhances liver metabolism, reduces inflammation, improves insulin signalling pathways, promotes positive adaptations in body composition, blood pressure, glucose metabolism, and lipid profiles	Mekari et al. (2020), Men et al. (2023), Hu et al. (2023), Zheng et al. (2020), Bo et al. (2023)
Sleep Quality	Improves sleep quality, reduces sleep onset latency, lowers wake after sleep onset.	Coswig et al. (2020), Labrin et al. (2023)
Quality of Life in Older Adults	Enhances body composition, insulin resistance, blood lipids, functional capacity, cardiorespiratory fitness, and quality of life compared to moderate-intensity continuous training (MICT), improves blood glucose, blood pressure, body mass index, and self-management efficacy	Rohmansyah et al. (2023), Juan and Xianyi (2023), Keating et al. (2020), Stern et al. (2023), Marriott et al. (2021)
Safety for Seniors with Chronic Conditions	Well-tolerated and beneficial, enhances functional movement, improves cardiorespiratory fitness, though specific effects need further exploration	Brockway et al. (2023), Ito (2022b), Marriott et al. (2021)
Functional Movement and Fall Risk	Improves lower limb strength, dynamic balance, subjective balance perception, can be a valuable supplement to fall prevention programs, though neuromuscular coordination patterns might vary	Elboim-Gabyzon et al. (2021), Donath et al. (2015), Falck et al. (2017)
Chronic Conditions Management	Improves functional movement and cardiorespiratory fitness, reduces the risk of chronic diseases like hypertension, diabetes, and chronic respiratory conditions	Juan and Xianyi (2023), Keating et al. (2020), Stern et al. (2023), Brockway et al. (2023), Ito (2022a)

## 4 Continuous aerobic training: the comprehensive benefits for health and aging

Continuous Aerobic Training (CAT) is a form of cardiovascular exercise characterized by sustained, steady-state activity performed at a moderate intensity over an extended period. Unlike interval training, which alternates between high and low intensities, CAT maintains a consistent pace that is typically between 60%–80% of an individual’s maximum heart rate. This training method is designed to improve aerobic capacity, endurance, and overall cardiovascular health by enhancing the efficiency of the heart, lungs, and circulatory system. Common activities for CAT include running, cycling, swimming, and brisk walking, which can be performed for durations ranging from 30 min to several hours, depending on the individual’s fitness level and training goals.

Recent research has highlighted the numerous benefits of CAT. It is particularly effective in increasing mitochondrial density and improving the oxidative capacity of muscles, which are crucial for enhanced endurance performance. Additionally, CAT has been shown to reduce the risk of chronic diseases such as cardiovascular disease, type 2 diabetes, and obesity (Sahin et al., 2020).

Furthermore, CAT is associated with various mental health benefits. Regular aerobic exercise has been linked to improved mood, reduced symptoms of depression and anxiety, and enhanced cognitive function. This is partly due to the release of endorphins and other neurotransmitters that promote a sense of wellbeing and mental clarity (Pan et al., 2019).

CAT is accessible to a wide range of fitness levels, making it an inclusive option for people of varying ages and abilities. Beginners can start with low-impact activities like walking or cycling at a comfortable pace and gradually increase intensity and duration as their fitness improves. Despite its many benefits, CAT should be complemented with other forms of exercise for a well-rounded fitness program. Strength training and flexibility exercises are important to ensure balanced physical development and prevent injuries. Moreover, varying workout routines can prevent the monotony that might come with continuous aerobic activities and can further enhance overall fitness by targeting different muscle groups and energy systems.

CAT offers extensive benefits for the aging population, significantly enhancing physical and mental health. CAT, which includes activities such as walking, cycling, and swimming at a moderate intensity for extended periods, is particularly effective in improving cardiovascular health. Studies have shown that aerobic exercise can lead to improvements in various health indicators such as blood pressure, blood lipids, body mass index, and blood oxygen saturation (Juan and Xianyi, 2023). Additionally, it has been found to enhance cardiopulmonary function, flexibility, and physical quality, optimizing activities of daily living in the elderly (Mei and Chang, 2023). Furthermore, aerobic exercise can increase submaximal exercise capacity, improve neuromuscular quality, and enhance walking distance in older individuals, thus positively impacting their overall fitness and mobility (Hou and Sun, 2022). Moreover, CAT has been linked to increased cerebral blood flow, decreased central arterial stiffness, and improved cognitive function, including memory and executive function, in older adults with or without mild cognitive impairment (Zhang, 2022). These findings collectively highlight the significant advantages of CAT in promoting healthy aging by addressing both physical and cognitive aspects.

Regarding metabolic health, CAT is instrumental in maintaining a healthy body weight and reducing the risk of chronic conditions such as diabetes and obesity. According to research in Diabetes Care (Colberg et al., 2016), consistent aerobic exercise improves insulin sensitivity and glycemic control, thereby lowering the risk of type 2 diabetes in older adults. Additionally, CAT supports weight management by increasing caloric expenditure and enhancing metabolic rate, which is vital for preventing obesity-related complications. Studies have shown that aerobic exercise in older adults with Diabetes Mellitus type 2 (DM2) leads to better glycemic control, decreased insulin resistance, improved pancreatic β-cell function, increased self-esteem, and a sense of wellbeing, ultimately translating into an improved quality of life (Ramírez-Alvarado, 2023).

CAT has positively impacted cognitive function in aging individuals. Research indicates that aerobic exercise, such as aerobics, can enhance cognitive abilities by stimulating the brain during physical movements, improving memory, and promoting overall brain health (Afradi, 2023). Regular aerobic exercise has been shown to reduce symptoms of depression and anxiety and improve overall mood. A meta-analysis published in JAMA Psychiatry (Schuch et al., 2016) highlighted that aerobic exercise significantly decreased depressive symptoms in older adults, suggesting its potential as an adjunct treatment for depression. Furthermore, aerobic exercise enhances cognitive function, which is essential for mitigating age-related cognitive decline. Recently, other studies have demonstrated that aerobic physical activity aimed at improving cardiorespiratory fitness can have beneficial effects on cognition in older adults without cognitive impairment, potentially improving cardiovascular fitness and cognitive function (Guadagni et al., 2020). Aerobic exercise has been found to modulate cerebral microcirculatory changes induced by aging, partly renormalizing microvascular perfusion and oxygenation in the brain, leading to improvements in cognitive function (Shin et al., 2023). Additionally, lifelong aerobic exercise training has been associated with higher cerebrovascular responsiveness to hypercapnia and cognitive stimuli and better total composite cognitive scores in older adults, highlighting the relationship between regular exercise and cognitive function in aging populations (Bliss et al., 2023).

CAT plays a crucial role in improving cardiovascular health as individual age. Research has shown that aerobic exercise training positively impacts various aspects of cardiovascular health, such as lipid profiles, apolipoproteins, and lipoprotein sub-fractions, which are key predictors of cardiovascular disease risk (Wood et al., 2023). Additionally, aerobic training has been found to enhance endothelial function, reduce arterial stiffness, and improve vasculature markers, ultimately reducing cardiovascular risk in older individuals (Kleinloog et al., 2022; Lake et al., 2022; Tanaka, 2019). Furthermore, engaging in long-term aerobic exercise has been shown to increase the level of circulating endothelial progenitor cells, which play a vital role in repairing damaged vascular endothelium and preventing cardiovascular events in middle-aged and older adults (Tao et al., 2023). Therefore, incorporating continuous aerobic training into one’s routine, especially as they age, can significantly contribute to maintaining cardiovascular health and reducing the risk of cardiovascular diseases. Additionally, aerobic exercise has been linked to a decrease in cerebrovascular resistance index (CVRi), an increase in cerebral blood flow (CBF), and improvements in cardiovascular and cerebrovascular function, which are essential for maintaining health and independence in older adults (Lake et al., 2022). Moderate-to-vigorous intensity aerobic exercise has been found to increase CBF and decrease central arterial stiffness in older adults, potentially preceding positive effects on brain structure and neurocognitive function, further contributing to overall wellbeing and longevity (R. Zhang, 2022).

Moreover, CAT is beneficial for musculoskeletal health: it helps in preserving muscle mass and bone density, which are critical for maintaining mobility and reducing the risk of falls and fractures. Research found that older adults who participated in regular aerobic exercise had higher bone mineral density and muscle strength, which are vital for preventing osteoporosis and sarcopenia. The low-impact nature of many aerobic activities also reduces the risk of injury, making it a safe and effective option for older adults (Laurin et al., 2019).

CAT has been shown to be beneficial in reducing the risk of age-related diseases. Studies have indicated that aerobic exercise interventions lead to improvements in various health indicators among the elderly, such as decreased detection rates of liver steatosis, overweight, and obesity, as well as better control of blood pressure, blood glucose, and blood lipid levels (Jianzhuang et al., 2022). Additionally, aerobic exercise has been linked to enhanced cardiovascular and cerebrovascular function, which can help mitigate age-related declines in everyday life and maintain independence in older adults (Lake et al., 2022). Furthermore, combining intermittent hypoxic-hyperoxic exposure with aerobic training has demonstrated positive effects on reducing systolic blood pressure in geriatric patients, highlighting the potential of such interventions in managing cardiovascular risk factors in the elderly (Behrendt et al., 2022). Regular physical activity, including aerobic training, plays a crucial role in preventing and treating vascular dysfunction and cardiometabolic diseases associated with aging, emphasizing the importance of exercise in promoting healthy aging and reducing the burden of age-related diseases (Tanaka, 2019).

Current limitations in understanding how CAT affects aging include the need for further research to determine the primary determinants of the BDNF response to aerobic training in seniors (Enette et al., 2017). Aerobic exercise can mitigate the physiological effects of aging and enhance active life expectancy by reducing the risk of chronic diseases and disabilities (Lazzer et al., 2018). Additionally, the relationship between aerobic exercise and mortality, particularly in the context of long-term endurance training, requires more in-depth mechanistic studies to elucidate how exercise influences cellular respiration and contributes to overall health and longevity (Koch and Britton, 2020). Further exploration is needed to understand how affective valence during aerobic exercise in older adults influences adherence and the long-term benefits of exercise on aging-related health outcomes (Smith-Ricketts et al., 2022). However, the sustainability and accessibility of CAT make it an excellent choice for long-term adherence. Its moderate intensity and variety of forms ensure that older adults can find activities that suit their preferences and physical capabilities, promoting consistent engagement. CAT offers a holistic approach to health maintenance and enhancement in older adults and plays a pivotal role in promoting healthy aging by improving cardiovascular and metabolic health, enhancing mental wellbeing, and preserving musculoskeletal function (see Table 3). Its moderate, sustained nature ensures it is safe and effective, making it a valuable component of a comprehensive health strategy for the elderly.

**TABLE 3:** CAT benefits on aging.

Benefit	Description	References
Mitochondrial Density and Oxidative Capacity	Improves muscle endurance and performance	Sahin et al. (2020)
Risk of Chronic Diseases	Lowers the risk of cardiovascular disease, type 2 diabetes, and obesity	Sahin et al. (2020)
Mental Health	Enhances mood, reduces symptoms of depression and anxiety, and improves cognitive function	Pan et al. (2019)
Cardiovascular Health	Enhances heart and lung efficiency, improves blood pressure, blood lipids, and blood oxygen saturation	Juan and Xianyi (2023), Wood et al. (2023)
Physical Quality in Elderly	Improves flexibility, neuromuscular quality, and walking distance, optimising activities of daily living	Mei and Chang (2023), Hou and Sun (2022)
Cognitive Function in Ageing	Increases cerebral blood flow, reduces central arterial stiffness, enhances memory and executive function	Zhang (2022), Afradi (2023)
Metabolic Health	Improves insulin sensitivity, glycemic control, supports weight management, and enhances metabolic rate	Colberg et al. (2016), Ramírez-Alvarado (2023)
Cognitive Function Enhancement	Stimulates brain, improves memory, and overall brain health, reduces symptoms of depression and anxiety	Guadagni et al. (2020), Schuch et al. (2016), Shin et al. (2023)
Musculoskeletal Health	Preserves muscle mass and bone density, reducing the risk of falls and fractures	Laurin et al. (2019)
Age-Related Diseases	Decreases liver steatosis, overweight, obesity, and improves blood pressure, blood glucose, and lipid levels	Jianzhuang et al. (2022)
Vascular Function and Cardiovascular Risk	Enhances endothelial function, reduces arterial stiffness, improves vascular markers, and increases endothelial progenitor cells	Kleinloog et al. (2022), Lake et al. (2022), Tanaka (2019), Tao et al. (2023)
Overall Health and Longevity	Mitigates physiological effects of ageing, reduces chronic disease risk, improves quality of life	Lazzer et al. (2018), Koch and Britton (2020)
Accessibility and Long-Term Adherence	Suitable for various fitness levels, promoting consistent engagement and long-term health benefits	Smith-Ricketts et al. (2022)
Cardiovascular and Cerebrovascular Function	Increases cerebral blood flow, decreases central arterial stiffness, and improves cognitive function	Lake et al. (2022), Zhang (2022)
Prevention of Osteoporosis and Sarcopenia	Regular aerobic exercise leads to higher bone mineral density and muscle strength	Laurin et al. (2019)
Cardiovascular Risk Factors	Combining intermittent hypoxic-hyperoxic exposure with aerobic training reduces systolic blood pressure	Behrendt et al. (2022)
Promotion of Healthy Ageing	Enhances physical and cognitive aspects, ensuring a holistic approach to ageing	Bliss et al. (2023), Tanaka (2019)

## 5 Discussion

High-Intensity Interval Training (HIIT) and Continuous Aerobic Training (CAT) are prominent exercise modalities that significantly impact aging, particularly in older adults. Understanding their differences and implications is crucial for developing effective exercise regimens aimed at enhancing longevity and quality of life in this demographic.

In recent research, Wang et al. have studied the systemic effects of CAT and HIIT, analyzing the differentially expressed (DE) miRNAs present in Extracellular Vesicles (EVs).

EVs are a family of lipid bilayer vesicles secreted by almost all cells, which contain bioactive molecules such as proteins, nucleic acids, and lipids that are involved in intercellular and inter-organ communication.

MiRNA represents a class of non-coding RNAs with a length of 20–25 nucleotides, which are involved in post-transcriptional regulation playing an important role in different physiological and pathological processes. EVs contain miRNAs; in fact, a recent study has demonstrated that miRNAs–EVs produced after exercise induce health-promoting processes such as cardiovascular protection and white adipose tissue browning (Di et al., 2020; Zhao et al., 2022).

In the work of Wang and coworkers, miRNA profiles of plasma EVs obtained from REST, CAT, and HIIT groups were analyzed. The authors found that 67 DE miRNAs (22 upregulated and 45 downregulated, SI-DE miRNAs) were identified in the CAT group compared to those in the REST group, while 13 DE miRNAs (7 upregulated and 6 downregulated) were identified in the HIIT group compared to those in the REST group.

In the CAT group, 23 pathways were found to be co-regulated by EV miRNAs and EV proteins, these pathways were involved in metabolism (lipid and sterol) and the maintenance of cellular homeostasis. In the HIIT group, 29 pathways were found to be co-regulated by EV miRNAs and EV proteins, these pathways were primarily associated with phospholipid metabolism, insulin secretion, and cellular physiological functions. These data further confirmed the overlapping and distinct biological roles of CAT and HIIT (Wang et al., 2025).

HIIT involves short bursts of intense exercise followed by rest or low-intensity periods. This form of training is typically characterized by its efficiency, requiring shorter time commitments than traditional forms of exercise. Conversely, CAT, also known as steady-state or endurance training, involves sustained, moderate-intensity exercise over a longer period, such as jogging, cycling, or swimming.

One of the most significant distinctions between HIIT and CAT is their effects on cardiovascular health. Studies have demonstrated that HIIT can produce superior improvements in cardiovascular fitness compared to CAT. A meta-analysis by Ramos et al. (2015) found that HIIT elicited greater enhancements in VO2 max, a key indicator of cardiovascular health, than CAT (Ramos et al., 2015). This is particularly relevant for older adults, as cardiovascular fitness is closely linked to longevity and reduced risk of cardiovascular diseases. HIIT’s effectiveness in improving cardiovascular function is attributed to its ability to induce greater cardiac output and oxygen utilization during intense periods of exercise, even in shorter durations (Fisher et al., 2015).

Another crucial aspect of HIIT is its impact on muscle strength and power. The high-intensity nature of HIIT workouts often involves resistance exercises or plyometrics, which can significantly improve muscle mass and function. This is particularly beneficial for older adults, as sarcopenia, or age-related muscle loss, is a common issue that can lead to decreased mobility and increased risk of falls and fractures. Studies such as those by Robinson et al. (2017) suggest that HIIT can stimulate muscle protein synthesis and promote hypertrophy, thereby counteracting the effects of sarcopenia more effectively than CAT (Robinson et al., 2017).

In terms of metabolic health, HIIT has shown remarkable benefits in improving insulin sensitivity and glucose metabolism. Older adults are at a higher risk of developing metabolic disorders such as type 2 diabetes. HIIT has been demonstrated to enhance insulin sensitivity significantly more than CAT, as shown in research by Cassidy et al. (2017) (Cassidy et al., 2017). This improvement is critical for older adults, as better insulin sensitivity helps regulate blood glucose levels, reducing the risk of diabetes and its associated complications. Additionally, Wewege et al. (2017) reported that HIIT could induce significant reductions in visceral fat, a risk factor for metabolic syndrome, which is highly prevalent among older adults (Wewege et al., 2017).

Conversely, CAT offers unique benefits that complement those of HIIT. CAT is particularly effective in enhancing aerobic capacity and endurance, essential for performing daily activities and maintaining independence in older age. Regular CAT has been associated with improvements in mitochondrial function and oxidative capacity of muscles, which are vital for sustained physical activity (Distefano and Goodpaster, 2018). Additionally, CAT is generally associated with lower injury risks than HIIT, making it a safer option for older adults with pre-existing health conditions or lower baseline fitness levels.

CAT also plays a significant role in mental health, which is crucial for overall wellbeing in older adults. Engaging in continuous aerobic activities like walking or cycling can have profound effects on mental health, reducing symptoms of depression and anxiety. The rhythmic and repetitive nature of CAT activities is believed to promote the release of endorphins, often called ‘feel-good’ hormones, which can enhance mood and cognitive function. Research by Gomes-Osman et al. (2018) supports the notion that aerobic exercise can stimulate neurogenesis and improve cognitive function, potentially reducing the risk of dementia and other cognitive impairments in older adults (Gomes-Osman et al., 2018). Similarly, Firth et al. (2018) found that CAT could increase hippocampal volume and be associated with better memory function (Firth et al., 2018).

Despite the numerous benefits of both HIIT and CAT, it is essential to consider individual differences and preferences when designing exercise programs for older adults. The high intensity of HIIT may not be suitable for everyone, particularly those with cardiovascular issues, joint problems, or low fitness levels. Therefore, a thorough health assessment and consultation with healthcare professionals are recommended before starting a HIIT program.

For older adults new to exercise or with significant health concerns, starting with CAT may be more appropriate. CAT can serve as a foundation, building baseline fitness and confidence before potentially incorporating HIIT elements. Gradual progression and careful monitoring are crucial to ensure safety and efficacy. Importantly, both forms of exercise can be tailored to individual capabilities to maximize benefits and minimize risks.

Moreover, a hybrid approach combining HIIT and CAT elements may offer the most comprehensive benefits. For example, an exercise regimen could include moderate-intensity aerobic activities for endurance, complemented by occasional high-intensity intervals to boost cardiovascular and metabolic health. This balanced approach can maximize the advantages of both training modalities while minimizing risks.

In addition to the physical benefits, social aspects of exercise should not be overlooked. Group activities, whether in the form of CAT or group HIIT classes, can provide social interaction and support, vital for mental health and adherence to exercise programs (Batrakoulis and Fatouros, 2022). Engaging in physical activities with peers can enhance motivation, enjoyment, and long-term commitment to an active lifestyle (Firth et al., 2016).

## 6 Conclusion

**FIGURE 1:**
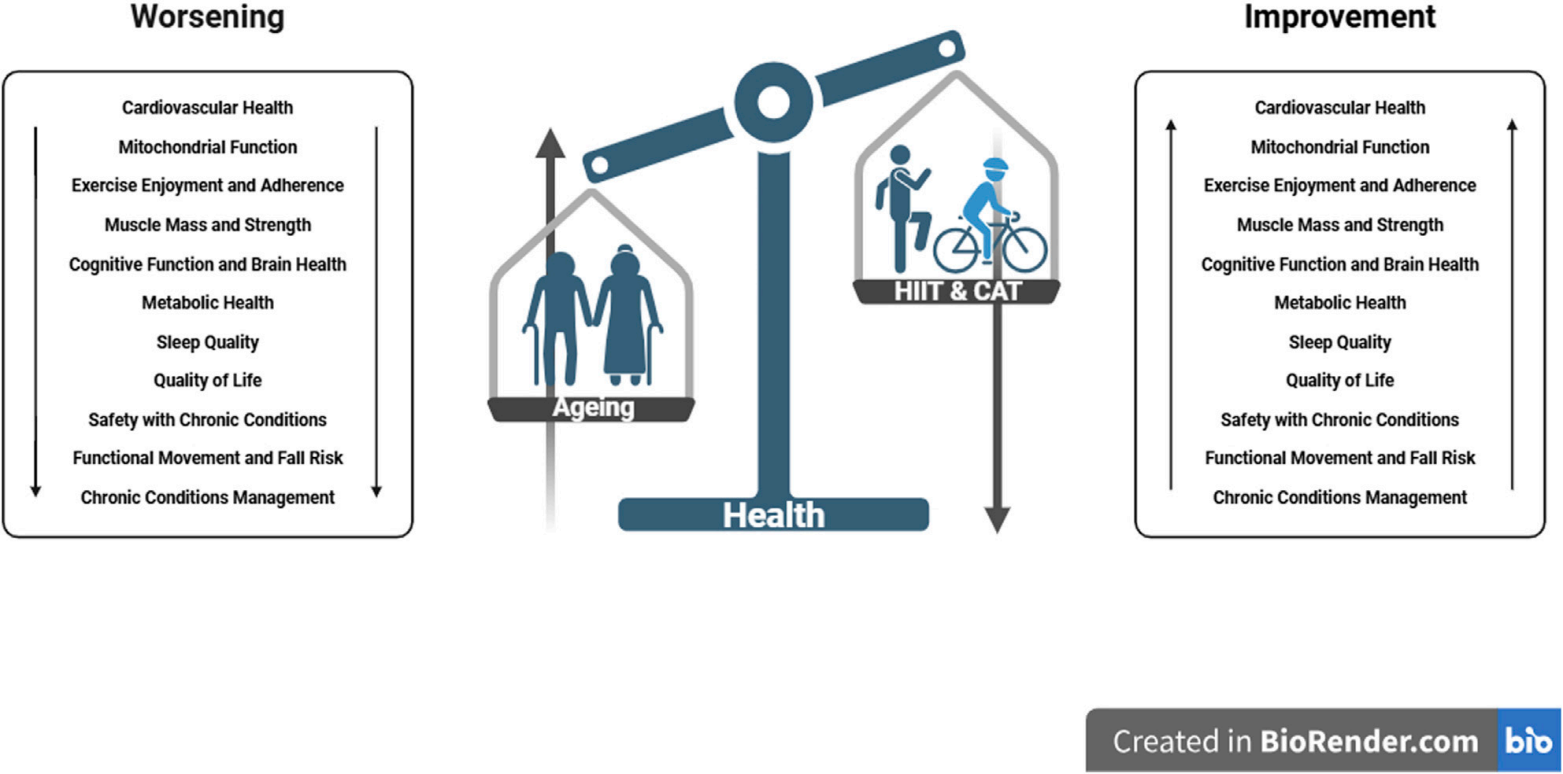
The figure shows the improvements due to HIIT and CAT on aging.

HIIT and Continuous Aerobic Training offer distinct and significant benefits for ageing populations. HIIT is particularly effective for improving cardiovascular fitness, muscle strength, and metabolic health, while CAT excels in enhancing aerobic capacity, endurance, and mental health. When designing exercise programs for older adults, it is essential to consider individual health status, preferences, and goals. A personalized and balanced approach that potentially combines elements of both HIIT and CAT, along with proper nutritional support and social engagement, is likely to yield the best outcomes for promoting health, longevity, and quality of life in older adults (Figure 1). Consulting with healthcare professionals before starting any new exercise regimen is crucial to ensure safety and appropriateness for individual health conditions.
